# Increased activated regulatory T cell subsets and aging Treg-like cells in multiple myeloma and monoclonal gammopathy of undetermined significance: a case control study

**DOI:** 10.1186/s12935-018-0687-8

**Published:** 2018-11-19

**Authors:** Ji-nuo Wang, Xin-xin Cao, Ai-lin Zhao, Hao Cai, Xuan Wang, Jian Li

**Affiliations:** Department of Hematology, Peking Union Medical College Hospital, Chinese Academy of Medical Sciences and Peking Union Medical College, Beijing, China

**Keywords:** Multiple myeloma, Monoclonal gammopathy of undetermined significance, Immune dysfunction, Regulatory T cell

## Abstract

**Background:**

Accumulating evidence have indicated that regulatory T cells (Tregs) play an essential role in T cell-mediated immune response and development of multiple myeloma (MM). CD4^+^FoxP3^+^ T cells are composed of three phenotypically and functionally distinct subpopulations: CD45RA^+^FoxP3^lo^ resting Tregs (rTregs), CD45RA^−^FoxP3^hi^ activated Tregs (aTregs) and CD45RA^−^FoxP3^lo^ non-suppressive T cells (non-Tregs). We aimed to clarify the frequency and function of these three subpopulations in newly diagnosed multiple myeloma and monoclonal gammopathy of undetermined significance (MGUS) patients. In addition, CD28^−^CD4^+^FoxP3^+^ Treg-like cell is a senescent regulatory T cell subset with partial suppressive function, which could be impaired during myelomagenesis.

**Methods:**

we examined 20 patients with MGUS, 26 patients with newly diagnosed MM and 18 healthy volunteers. Flow cytometric analysis in peripheral blood and bone marrow was performed for frequency study. The immunosuppressive function of Treg subsets was assessed by their ability to suppress the proliferation of responder cells in co-culture. Concentration of cytokine from the culture supernatants of proliferation assay was measured using ELISA.

**Results:**

The proportion of activated Tregs in CD4^+^ T cells was significantly higher in MGUS and MM patients than healthy controls (*P *= 0.01, *P *< 0.001) in both PB and BM; while the proportion of rTregs in MGUS, MM patients was significantly lower than that of controls (*P *= 0.02, *P *< 0.01) only in BM. There was no significant difference in frequencies of non-Tregs from MGUS to MM patients with normal controls (*P *= 0.14, *P *= 0.88). Significant increase in PB and BM Treg-like cells was observed in MGUS and MM cohort compared with healthy controls (*P *< 0.01, *P *< 0.01). Treg-like cells in MM patients were significantly higher than those in MGUS patients (*P *< 0.01). The inhibition rate of aTreg in bone marrow of MM patients was significantly higher than that of rTreg (*P *< 0.01), while the inhibition rate of non-Treg was significantly lower than that of rTreg cells (*P *< 0.01). Functional assays revealed the suppressive and secretory abilities of three Treg subsets were intact in MM patients.

**Conclusions:**

In summary, aTregs and aging Treg-like cells were quantitatively altered in MGUS and MM patients, which might be associated with disease progression and prognosis.

**Electronic supplementary material:**

The online version of this article (10.1186/s12935-018-0687-8) contains supplementary material, which is available to authorized users.

## Background

Multiple myeloma (MM) is a common hematologic malignancy characterized by renal insufficiency, osteolytic lesions, anemia and hypercalcemia [1]. Almost all MM cases were progressed from a premalignant condition called monoclonal gammopathy of undetermined significance (MGUS) [2]. So far, the pathogenesis of myeloma is not yet clear [3]. Changes in tumor microenvironment and genetic alterations synergically promote disease occurrence and progression [4, 5]. The immune cells in the tumor microenvironment, such as T and B cells, play a crucial part in myelomagenesis. As the major suppressors of immune responses, Tregs can regulate the proliferation and function of other immune cells, such as CD4^+^ and CD8^+^ T cells, natural killer cells, dendritic cells [6, 7]. Tregs can be further classified into three subpopulations based on their different phenotypes and functions [8]. The three distinct subpopulations are as follows: (1) CD45RA^+^FoxP3^lo^ resting Treg cells (rTregs) (2) CD45RA^−^FoxP3^hi^ activated Treg cells (aTregs), both of which are suppressive in vitro; (3) non-suppressive CD45RA^−^FoxP3^lo^ T cells (non-Tregs) which can secret immunoregulatory cytokines, such as IL-10, TGF-β and so on.

Several studies have shown that CD4^+^ Tregs are increased and functionally immunosuppressive in the peripheral blood of MM patients [9–11]. However, these results were strongly debated due to different gating strategies of Treg, lack of analysis based on Treg cell subsets. Besides, using peripheral blood rather than bone marrow as study subject cannot truly interpret the real size and function of Treg pools in tumor microenvironment since bone marrow is a possible priming site for T cell responses which represents the immediate tumor environment of myeloma [12, 13]. Therefore, we conducted our study based on precisely Treg subset analysis in both peripheral blood and bone marrow from MGUS to MM patients.

CD28 is the main co-stimulatory molecule of T cells and plays a key role in proliferation, differentiation and activation of T cells with CD3 synergistically [14]. Prolonged antigen stimulation and T cell senescence may cause the downregulation of CD28, leading to T cell anergy [15]. Recent studies found that immune abnormalities in MM patients are inseparable from functional failure caused by T cell aging [16], especially defective expression of CD28 in T cells, which has close relationship with clinical stage, disease progression and prognosis [17, 18]. CD4^+^CD28^−^FoxP3^+^ Treg-like cell is a novel senescent regulatory T cell subset with impaired suppressive function and early aging features [19]. In some connective tissue diseases, for example rheumatoid arthritis (RA), CD28^−^ Treg-like cells revealed an increased number and abnormal cytokine secretion which might contribute to pathogenic immune response. Thus we assumed these aging Treg-like cells might participate in immune dysfunction during myelomagenesis to some extent.

In this study, we performed a comprehensive analysis to investigate the frequencies and function of Treg cell subsets and aging Treg-like cells in both peripheral blood and bone marrow of newly diagnosed MM and MGUS patients, trying to provide new ideas for immunodeficiency of multiple myeloma.

## Methods

### Patients and healthy donors

In this study, patients were included after signing informed consent form, and the study was approved by Peking Union Medical College Hospital research ethics committee. A total of 20 MGUS and 26 untreated MM patients were recruited for this study. A group of 18 healthy adults (10 males and 8 females) who did not have any systemic disorders was studied in parallel and used as normal controls. Mononuclear cells from additional 8 MM patients and additional 3 volunteers were used for immunosuppressive assays.

### Flow cytometry analysis

PB and BM samples were collected and used for analysis within 24 h. Both PB and BM were lysed to remove erythrocytes using BD lysing solution (BD Biosciences, USA) according to manufacturer’s instructions. After lysis of erythrocytes, 1 × 10^6^ cells were labeled with the following fluorochrome conjugated monoclonal antibodies (BD Biosciences, USA): Fluorescein isothiocyanate (FITC) anti-hCD4 (clone RPA-T4), phycoerythrin-cyanin (PE-Cy™7) anti-hCD45RA (clone HI100), APC-Vio770 anti-hCD28 (clone 15E8) and Peridinin Chlorophyll Protein Complex-Cy™5.5 (PerCP-Cy™5.5) anti-hCD25 (clone M-A251), and incubated at 4 °C for 20–30 min.

Then, cells were permeabilized according to eBioscience recommendations (eBioscience, USA). Finally, cells were labeled with anti-hFoxP3 (clone 3G3) conjugated with allophycocyanin (APC) from Miltenyi Biotec (Germany) and incubated at 4 °C for 30–40 min. All prepared samples were measured on BD FACS Canto II. FlowJo software (Version 10.0, TreeStar) was used for analysis of the cytometric data. At least 50,000 events were acquired from each sample.

### Definition of activated Treg, resting Treg and non-suppressive T cells

As in the previous study [8], we defined activated Treg (aTreg) as CD4^+^CD45RA^−^FoxP3^hi^ or CD4^+^CD45RA^−^CD25^++^ T cells, resting Treg (rTreg) as CD4^+^CD45RA^+^ FoxP3^lo^ or CD4^+^CD45RA^+^CD25^+^ T cells and non- suppressive T cells (non-Treg) as CD4^+^CD45RA^−^FoxP3^lo^ or CD4^+^CD45RA^−^CD25^+^ T cells (Additional file 1: Figure S1). Similarly, we defined aging Treg cells as CD4^+^CD28^−^FoxP3^+^ based on previous findings [19] (Additional file 2: Figure S2).

### Isolation of T regulatory cell subsets

9 ml blood samples were collected and used for analysis within 12 h. At first, mononuclear cells were isolated by Ficoll-Hypaque density gradient centrifugation (GE Healthcare company, USA), then resuspended in PBS supplemented with 1% penicillin and streptomycin (Sigma, USA) at a concentration of 1 × 10^6^ cells/ml. To isolate Treg cell subsets, purified mononuclear cells were labeled with PE anti-hCD4 (clone RPA-T4), APC anti-hCD25 (clone M-A251) and PE-Cy™7 anti-hCD45RA (clone HI100). Then, these labeled cells were sorted using a BD FACS Aria™ Cell Sorter (BD Bioscience, USA) into four different subpopulations as described previously [8, 20]: CD4^+^CD25^+^CD45RA^−^, CD4^+^CD25^+^CD45RA^+^, CD4^+^CD25^++^CD45RA^−^, and CD4^+^CD25^−^ fractions sorted as responder cells. Purity of sorted cells was 95% for all samples. The CD25^++^ gate was adjusted to contain CD4^+^ T cells that express CD25 more brightly than CD4^+^CD25^+^ cells.

### Assessment of T regulatory cells subsets immunomodulatory function

#### Cell stimulation and suppression assay

RPMI 1640 medium supplemented with 10% fetal bovine serum, 100 IU/ml penicillin and 100 mg/ml streptomycin (Sigma, USA) was used for T cell cultures. For the carboxyfluorescein diacetate succinimidyl ester (CFSE) dilution assay, CD4^+^CD25^−^ responder T cells were labeled with 1 mmol/ml CFSE (Invitrogen, USA) for 20 min at 37 °C. 1 × 10^4^ CD4^+^CD25^−^ T cells were co-cultured in the presence or absence of sorted Treg subsets cells (aTregs, rTregs or non-Tregs) for assessing their suppressive capacity at 1:1 ratio. Subsequently, mixed T cell cultures were stimulated with anti-CD3/CD28 coated beads (cell: beads = 1:1) (Dynabeads human T-activator CD3/CD28, Life Technologies, USA) in 96-well plates for 90–96 h. Proliferation of CFSE-labeled cells was analyzed by flow cytometry. Suppression percentage = (number of proliferating CFSE-labeled responder cells when co-cultured with suppressor cells/number of proliferating responder cells when cultured alone) × 100%. FlowJo software (Version 10.0, TreeStar) was used for analysis of the cytometric data.

#### Cytokine determination

Concentration of IL-10 from the co-culture supernatants of proliferation assays were measured using Human IL-10 ELISA^pro^ Kit (Mabtech biotec, Sweden) according to the manufacturer’s instructions.

### Statistical analysis

Data were expressed in median and range percentages for Treg cells and their subsets. Non-parametric analyses were used including Mann–Whitney U test and Kruskal–Wallis test to evaluate the difference between two and more independent groups. To evaluate the difference between groups, *P* value<0.05 was considered as significant.

## Results

### Frequency of aTregs, rTregs and non-Tregs among CD4^+^ T cells in Peripheral Blood

**Fig. 1:**
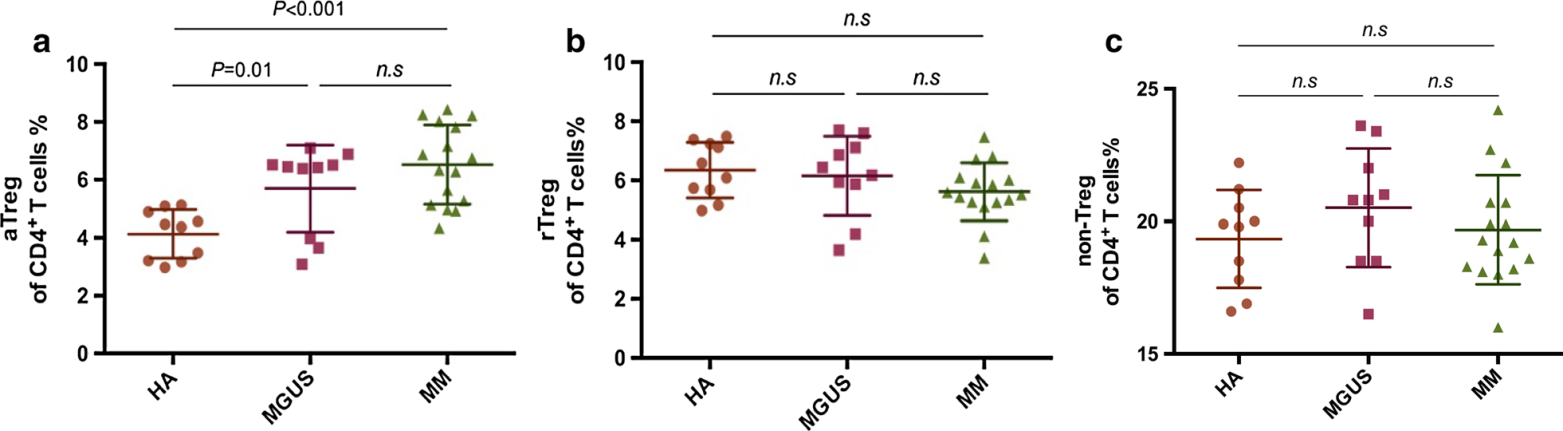
The proportion of Treg subsets in Peripheral Blood. Scattergrams show proportion of aTregs (**a**), rTregs (**b**) and non-Tregs (**c**) in PB from healthy adults (HA, n = 10), MGUS patients (n = 10) and myeloma patients (MM, n = 16). Mann–Whitney U test was used for statistical analysis

Quantification analysis showed that PB aTregs among CD4^+^ T cells were notably elevated in MGUS (5.70 ± 1.50%, n = 10, *P* < 0.01) and MM patients (6.52% ± 1.37%, n = 16, *P* < 0.0001) compared with healthy adults (4.13% ± 0.84%, n = 10), while there was no difference between MGUS and MM group (*P *= 0.16) (Fig. 1a). The frequency of rTregs among CD4^+^ T cells did not show any significance in MGUS patients (6.16% ± 1.34%, *P *= 0.72) and MM patients (5.69% ± 0.98%, *P *= 0.074) against healthy controls (6.35% ± 0.94%) (Fig. 1b). No significant difference in the frequency of non-Tregs among CD4^+^ T cells was observed among MGUS patients (19.34% ± 2.24%, *P *= 0.22) and MM patients (19.68% ± 2.05%, *P *= 0.67) compared with healthy adults (20.51% ± 1.84%) (Fig. 1c).

### Frequency of aTregs, rTregs and non-Tregs among CD4^+^ T cells in Bone Marrow

**Fig. 2:**
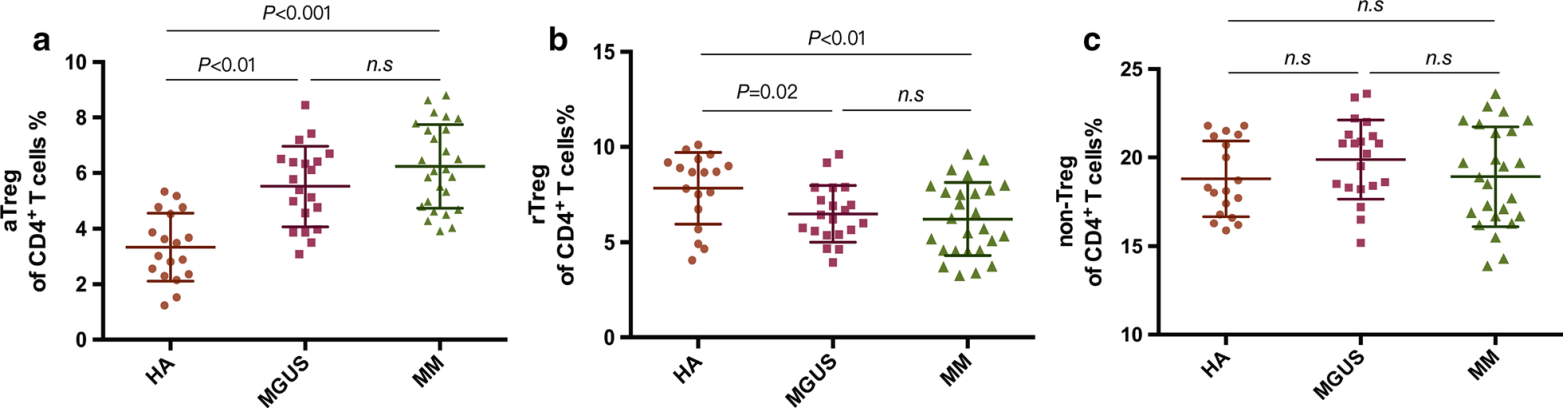
The proportion of Treg subsets in Bone Marrow. Scattergrams show proportion of aTreg (**a**), rTreg (**b**) and non-Treg (**c**) in BM from healthy adults (HA, n = 18), MGUS patients (n = 20) and newly diagnosed myeloma patients (MM, n = 26). Mann–Whitney U test was used for statistical analysis

Similar with PB, the frequency of BM aTregs among CD4^+^ T cells was dramatically higher in MGUS (5.52% ± 1.45%, n = 20, *P* < 0.0001) and MM patients (6.24% ± 1.51%, n = 26, *P* < 0.0001) than healthy adults (3.34% ± 1.23%, n = 18), whereas there was no difference between MGUS and MM group (*P *= 0.11) (Fig. 2a). Unlike PB results, significant decrease in BM rTreg cells was observed in MGUS (6.49% ± 1.48%, *P *= 0.02) cohort compared to healthy adults (7.83% ± 1.87%), and even decrease in MM patients (6.22% ± 1.91%, *P *= 0.009) (Fig. 2b). Non-Tregs among CD4^+^ T cells did not differ among patients with MGUS (19.88% ± 2.24%, *P *= 0.136), with untreated myeloma patients (18.92% ± 2.81%, *P *= 0.22) and healthy adults (18.79% ± 2.13%) (Fig. 2c).

### Frequency of aging Treg-like cells among CD4^+^ T cells in peripheral blood and bone marrow

**Fig. 3:**
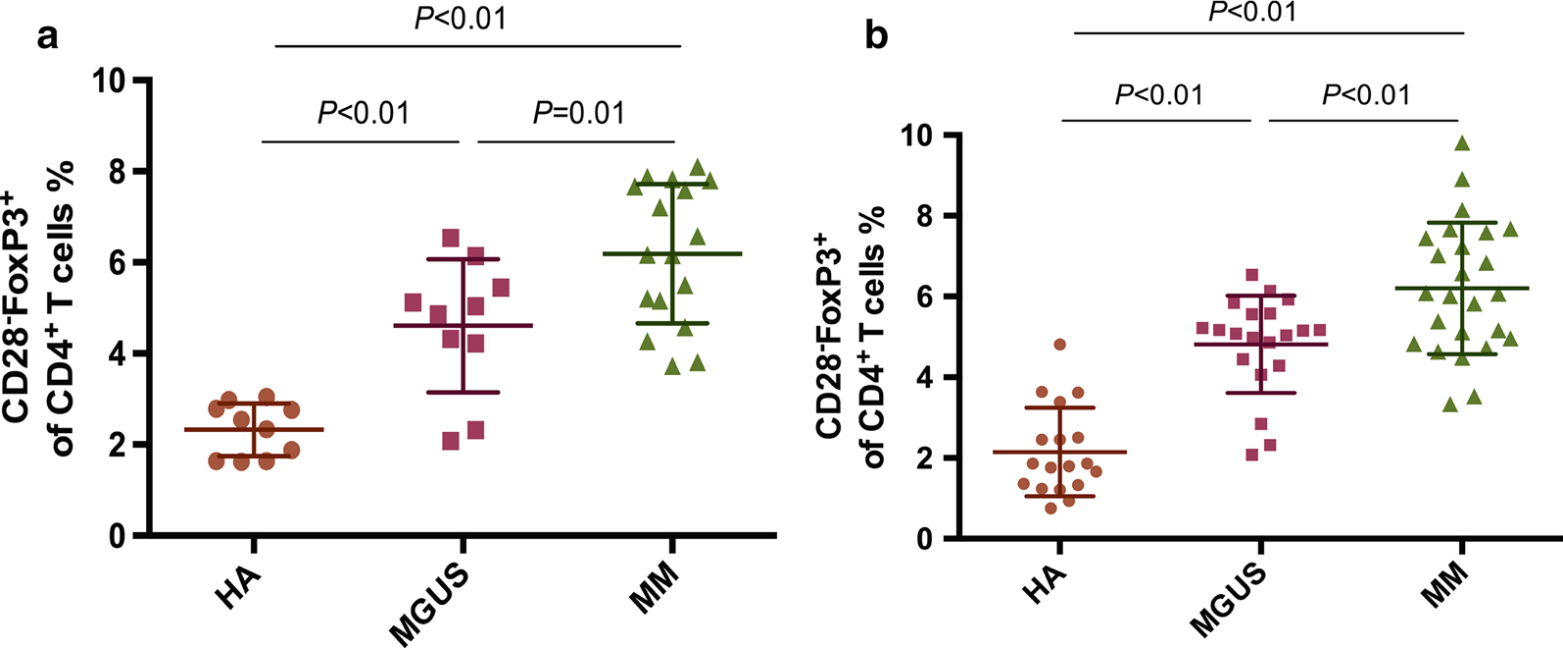
The proportion of Treg-like cells. Scattergrams show proportion of aging Treg-like cells in PB (**a**) and BM (**b**) from healthy adults (HA), MGUS and MM patients. Mann–Whitney U test was used for statistical analysis

In MGUS and MM patients but not in controls, we observed a FoxP3^+^ T cell subset lacking the expression of CD28. In PB, the proportion of circulating CD4^+^CD28^−^FoxP3^+^ Treg-like cells among CD4^+^ T cells significantly increased in MGUS patients (4.61% ± 1.46%, n = 10, *P *= 0.0002) and untreated myeloma patients (6.19% ± 0.1.58%, n = 16, *P *< 0.0001) compared to healthy individuals (2.33% ± 0.58%, n = 10); the frequency of Treg-like cells in MM patients was even remarkably higher than those in MGUS patients (*P *= 0.014) (Fig. 3a). Similarly, in BM, the proportion of Treg-like cells among CD4^+^ T cells in MGUS (4.82% ± 1.20%, n = 20, *P *< 0.0001) was notably higher than healthy controls (2.15% ± 1.10%, n = 18); in MM group, the proportion of Treg-like cells also showed a notable increase (6.20% ± 1.63%, n = 26) compared with MGUS cohort (*P *= 0.0027) and healthy adult group (*P *< 0.0001) (Fig. 3b).

### Suppressive function of Treg subsets in MM

**Fig. 4:**
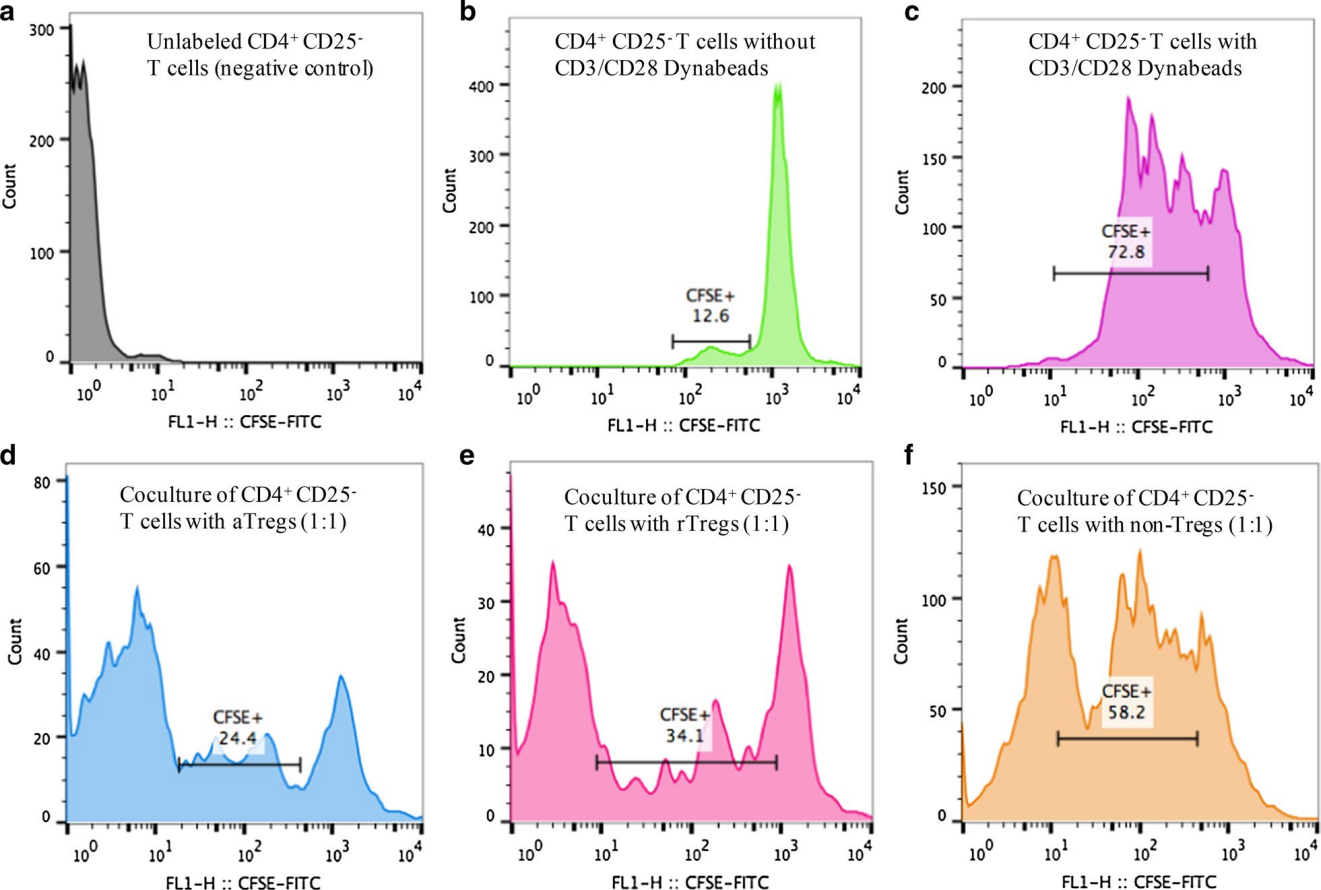
In vitro suppressive function assay from bone marrow of newly diagnosed MM patient. Representative curves are shown for a MM patient.** a** Negative control: unlabeled CD4^+^ CD25^−^ T cells alone.** b** Positive control 1: CFSE-labeled CD4^+^ CD25^−^ T cells without anti-CD3/CD28 beads.** c** Positive control 2: CFSE-labeled CD4^+^ CD25^−^ T cells with anti-CD3/CD28 beads.** d** aTreg group: CFSE-labeled CD4^+^ CD25^−^ T cells cocultured with aTreg at a ratio of 1:1.** e** rTreg group: CFSE-labeled CD4^+^ CD25^−^ T cells cocultured with rTreg at a ratio of 1:1.** f** non-Treg: CFSE-labeled CD4^+^ CD25^−^ T cells cocultured with non-Treg at a ratio of 1:1

**Fig. 5:**
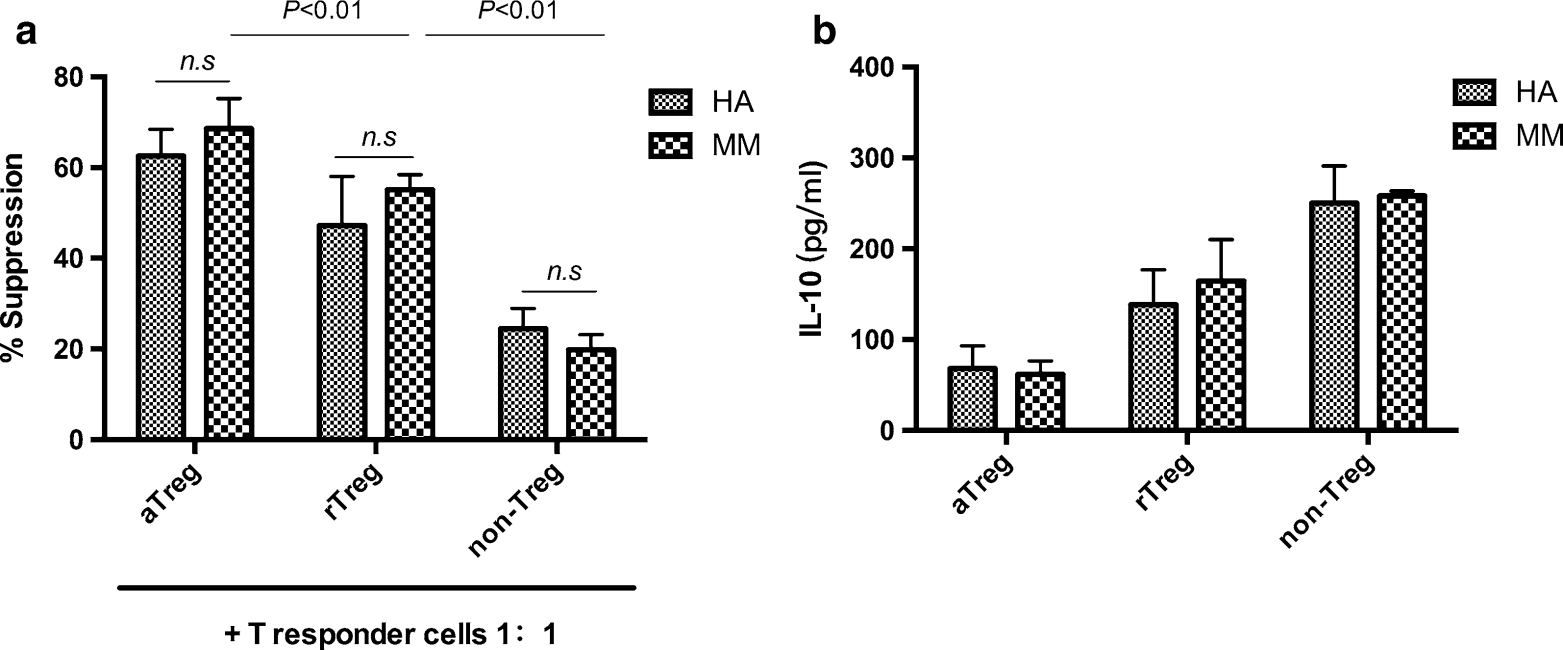
The immune function of Treg subsets in untreated myeloma patients.** a** The immunosuppressive function. 8 MM patients and 3 healthy volunteers were tested for immunosuppressive function by CFSE proliferation assay. Kruskal–Wallis test was used for statistical analysis.** b** The IL-10 secretory function. 2 out of 8 MM patients and 2 out of 3 healthy volunteers were tested the IL-10 levels by ELISA from the supernatants of co-culture

We assessed three Treg subsets respectively from 8 newly diagnosed MM patients and 3 healthy Adults for their suppressive function against responder cells (CD4^+^ CD25^−^ effective T cells) (Additional file 3: Table S1). Proliferation of CD4^+^CD25^−^ cells was inhibited in the presence of Treg cells to some extent, and inhibition was observed in subset-dependent manner (Fig. 4). The inhibition rate of aTreg cells (68.6% ± 6.7%) in the bone marrow of newly diagnosed MM patients was significantly higher than that of rTreg (55.1% ± 3.3%, *P *= 0.0002) and the inhibition rate of non-Treg cells (19.8% ± 3.4%) was significantly lower than that of rTreg cells (*P *< 0.0001). The inhibition rates of aTregs (*P *= 0.21), rTregs (*P *= 0.078) and non-Tregs (*P *= 0.089) in healthy controls were no difference from those in MM patients (Fig. 5a).

### The level of IL-10 in co-cultures of Treg subsets and responder cells

Measuring the level of IL-10 in supernatants from suppression assay co-cultures (responder cells: aTreg/rTreg/non-Treg = 1:1) revealed that the level of cytokine secreted by Tregs did not differ from healthy control subjects, respectively. The level of IL-10 secreted by non-Tregs in untreated MM patients was observationally higher than that of aTregs and rTregs (Fig. 5b).

## Discussion

Treg cells play an essential role in maintaining immunological homeostasis and exert major immunosuppressive activity. Elevated FoxP3 expression and Treg cell expansion are generally considered to poor prognosis markers in various cancers, including breast cancer, gastric malignancies, lymphoma and so on [21, 22]. FoxP3^+^ regulatory T cells could be divided into three different subpopulations: activated Treg cells (aTregs), resting Treg cells (rTregs) and non-suppressive Treg cells (non-Tregs) [8, 23]. rTreg is generated in the thymus and remain a resting state. After being stimulated by various antigens and activation signals in the body, it can be transformed into a terminally differentiated state called aTreg cell which has a stronger immunosuppressive function than rTreg cell [24]. In addition, Tregs contain a kind of non-Treg cells that have no immunosuppressive effect but can exert immunoregulatory function by secreting proinflammatory cytokines (IL-17, IL-10, TGF-β, etc.) [25].

Our study found that, no matter in PB or BM, the frequency of aTregs increased in newly diagnosed MM and MGUS patients, which indicated severe immunosuppressive state. However, changes of rTreg cells in peripheral blood and bone marrow of patients with MM or MGUS were not consistent. In MGUS and MM cohort, the proportion of rTreg cells in peripheral blood gradually decreased, which corresponded to the increasing proportion of aTreg cells. It may be explained that rTregs were transformed into aTregs by tumor antigen stimulation [26]. While in BM, the extent of rTreg decline in MGUS and newly diagnosed MM patients was more pronounced than in peripheral blood, suggesting bone marrow is more sensitive to reflect disease severity and progression. It’s worth mentioning that the proportion of aTreg cells in MGUS patients was significantly higher than that in control. Similarly, in the bone marrow of patients with MGUS, we also found that the proportion of rTreg cells was significantly lower than that of healthy people, indicating that there were already immunologic abnormalities in patients with precancerous lesions of myeloma. These results, from a side, support Dhodapkar’s view that patients in MGUS phase have experienced changes in the tumor microenvironment, including immune cells, osteoclasts, and stromal cells [27]. However, due to the short duration of our projects and limited samples, it was difficult to further analyze MM and MGUS patient based on risk stratification and disease severity.

For functional study, we found the inhibition rate of aTregs and rTregs on effective T cells was slightly higher than that of healthy controls, indicating the immune suppressive ability of Treg subsets in NDMM patients was basically normal. In addition, our results showed that the ability of Treg cell subsets to secrete IL-10 in untreated myeloma patients was not different from that of healthy controls. These results were partially consistent with previous studies [10, 28] that immunosuppressive function of CD4^+^CD25^hi^FoxP3^+^ cells in MM patients was intact compared with healthy controls.

In our study, we noticed that the proportion of CD28^−^ aging Treg-like cells in PB and BM from MGUS and MM patients was significantly higher than that of healthy controls, and gradually increased with disease progression. This also corresponds to our result of Treg subsets, suggesting that patient’s immunologic state is deteriorating during progression from MGUS to MM. The significant difference between MGUS patients and healthy controls revealed that in the early stage of myelomagenesis, phenotypic abnormalities of immune cells have appeared in the body of patients, leading to cellular senescence and functional failure.

## Conclusion

CD4^+^CD45RA^−^FoxP3^hi^ aTreg cells and CD4^+^CD28^−^FoxP3^+^ aging Treg-like cells were quantitatively impaired both in MGUS and newly diagnosed myeloma patients. These findings could provide new insight into the dynamics of CD4^+^FoxP3^+^ T cells and their role in the pathogenesis of multiple myeloma.

## Additional files

**Additional file 1.** The gating strategy of Treg subsets. Phenotype of Treg cell subsets: resting Treg cells, activated Treg cells and non-Treg cells. (A) PBMCs or BMMCs were gated on FSC, SSC and analyzed for lymphocytes. (B) Percentages of CD4^+^ T cells gated on CD4 and SSC. (C) Three subsets of CD4^+^ T cells are defined by the expression of CD45RA and FoxP3: CD45RA^+^FoxP3^lo^ cells, CD45RA-FoxP3^hi^ cells, CD45RA-FoxP3^lo^ cells; Representative dot plots are shown for an untreated MM patient.

**Additional file 2.** The gating strategy of CD28^−^ Treg-like cells. Phenotype of Treg-like cell subsets. (A) PBMCs or BMMCs were gated on FSC, SSC and analyzed for lymphocytes. (B) Percentages of CD4^+^ T cells gated on CD4 and SSC. (C) Percentages of CD4^+^FoxP3^+^ cells gated on FoxP3 and SSC. (D) Percentages of CD4^+^CD28^−^FoxP3^+^ cells gated on CD28 and FSC. Representative dot plots are shown for an untreated MM patient.

**Additional file 3.** The suppressive percentage of Treg subsets from MM patients and healthy volunteers.

## Abbreviations

MM: Multiple myeloma
MGUS: Monoclonal gammopathy of undetermined significance
Treg: regulatory T cell
aTreg: activated Treg
rTreg: resting Treg
non-Treg: non-suppressive Treg
PB: peripheral blood
BM: bone marrow
ELISA: enzyme-linked immuno sorbent assay
TGF-β: transforming growth factor-β
IL-10: interleukin-10
RA: rheumatoid arthritis
