# Supplementary material for: Increased activated regulatory T cell subsets and aging Treg-like cells in multiple myeloma and monoclonal gammopathy of undetermined significance: a case control study
Source: Cancer Cell Int. 2018 Nov 19;18:187. doi: 10.1186/s12935-018-0687-8 (PMC6245875; doi:10.1186/s12935-018-0687-8)
Supplement: Supplementary file 1 — Additional file 1. The gating strategy of Treg subsets. Phenotype of Treg cell subsets: resting Treg cells, activated Treg cells and non-Treg cells. (A) PBMCs or BMMCs were gated on FSC, SSC and analyzed for lymphocytes. (B) Percentages of CD4+ T cells gated on CD4 and SSC. (C) Three subsets of CD4+ T cells are defined by the expression of CD45RA and FoxP3: CD45RA+FoxP3lo cells, CD45RA-FoxP3hi cells, CD45RA-FoxP3lo cells; Representative dot plots are shown for an untreated MM patient. [file 12935_2018_687_MOESM1_ESM.pdf]

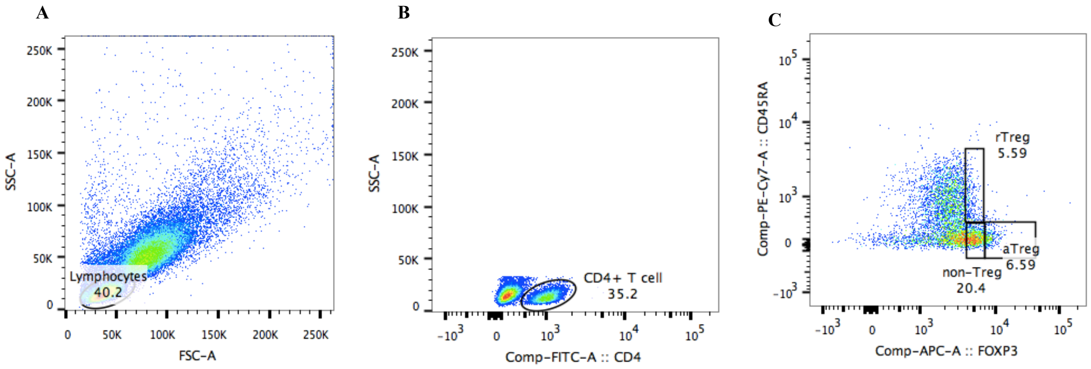

Additional file 1: Figure S1. The gating strategy of Treg subsets. Phenotype of Treg cell subsets: resting Treg cells, activated Treg cells and non-Treg cells. (A)PBMCs or BMMCs were gated on FSC, SSC and analyzed for lymphocytes. (B) Percentages of CD4$^+$ T cells gated on CD4 and SSC. (C) Three subsets of CD4$^+$ T cells are defined by the expression of CD45RA and FoxP3: CD45RA$^+$FoxP3^{lo} cells, CD45RA$^-$FoxP3^{hi} cells, CD45RA$^-$FoxP3^{lo} cells; Representative dot plots are shown for an untreated MM patient.
