# Supplementary material for: Increased activated regulatory T cell subsets and aging Treg-like cells in multiple myeloma and monoclonal gammopathy of undetermined significance: a case control study
Source: Cancer Cell Int. 2018 Nov 19;18:187. doi: 10.1186/s12935-018-0687-8 (PMC6245875; doi:10.1186/s12935-018-0687-8)
Supplement: Supplementary file 2 — Additional file 2. The gating strategy of CD28− Treg-like cells. Phenotype of Treg-like cell subsets. (A) PBMCs or BMMCs were gated on FSC, SSC and analyzed for lymphocytes. (B) Percentages of CD4+ T cells gated on CD4 and SSC. (C) Percentages of CD4+FoxP3+ cells gated on FoxP3 and SSC. (D) Percentages of CD4+CD28−FoxP3+ cells gated on CD28 and FSC. Representative dot plots are shown for an untreated MM patient. [file 12935_2018_687_MOESM2_ESM.pdf]

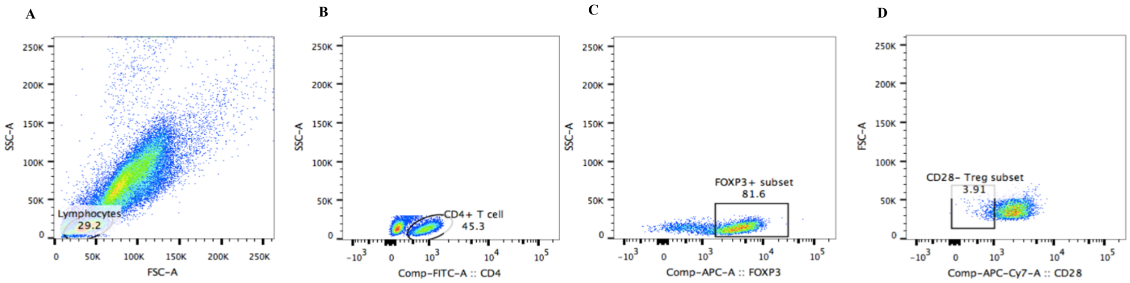

Additional file 2: Figure S2. The gating strategy of $CD28^-$ Treg-like cells. Phenotype of Treg-like cell subsets. (A) PBMCs or BMMCs were gated on FSC, SSC and analyzed for lymphocytes. (B) Percentages of $CD4^+$ T cells gated on CD4 and SSC. (C) Percentages of $CD4^+FoxP3^+$ cells gated on FoxP3 and SSC. (D) Percentages of $CD4^+CD28^-FoxP3^+$ cells gated on CD28 and FSC. Representative dot plots are shown for an untreated MM patient.
