# Supplementary material for: Increased activated regulatory T cell subsets and aging Treg-like cells in multiple myeloma and monoclonal gammopathy of undetermined significance: a case control study
Source: Cancer Cell Int. 2018 Nov 19;18:187. doi: 10.1186/s12935-018-0687-8 (PMC6245875; doi:10.1186/s12935-018-0687-8)
Supplement: Supplementary file 3 — Additional file 3. The suppressive percentage of Treg subsets from MM patients and healthy volunteers. [file 12935_2018_687_MOESM3_ESM.pdf]

Additional file 3: Table S1. The suppressive percentage* of Treg subsets from MM patients and healthy volunteers

MM	aTreg(%)	rTreg(%)	non-Treg (%)	HA	aTreg(%)	rTreg(%)	non-Treg (%)
01	66.5	49.7	20.1	01	68.2	59.7	20.3
02	67.7	59.1	16.8	02	63.1	41.1	24.0
03	66.0	55.5	21.2	03	56.5	40.9	29.2
04	69.0	52.1	20.1				
05	73.6	59.2	19.2				
06	56.8	53.3	20.0				
07	68.5	56.1	14.8				
08	80.3	56.1	26.4				

*Suppression percentage = (number of proliferating CFSE-labeled responder cells when co-cultured with suppressor cells/ number of proliferating responder cells when cultured alone) x100%.
